# Maladaptation of U.S. corn and soybeans to a changing climate

**DOI:** 10.1038/s41598-021-91192-5

**Published:** 2021-06-11

**Authors:** Chengzheng Yu, Ruiqing Miao, Madhu Khanna

**Affiliations:** 1 Department of Agricultural and Consumer Economics, University of Illinois at Urbana-Champaign, Urbana, IL USA; 2 Department of Agricultural Economics and Rural Sociology, Auburn University, Auburn, AL USA

**Keywords:** Climate sciences, Environmental social sciences

## Abstract

We quantify long-run adaptation of U.S. corn and soybean yields to changes in temperature and precipitation over 1951–2017. Results show that although the two crops became more heat- and drought-tolerant, their productivity under normal temperature and precipitation conditions decreased. Over 1951–2017, heat- and drought-tolerance increased corn and soybean yields by 33% and 20%, whereas maladaptation to normal conditions reduced yields by 41% and 87%, respectively, with large spatial variations in effects. Changes in climate are projected to reduce average corn and soybean yields by 39–68% and 86–92%, respectively, by 2050 relative to 2013–2017 depending on the warming scenario. After incorporating estimated effects of climate-neutral technological advances, the net change in yield ranges from (−)13 to 62% for corn and (−)57 to (−)26% for soybeans in 2050 relative to 2013–2017. Our analysis uncovers the inherent trade-offs and limitations of existing approaches to crop adaptation.

Concerns about the impact of climate change on agriculture have led to a growing interest in adapting crops to the increased frequency of deleterious weather events such as extreme heat and drought^1–5^. Numerous advances have been made in crop cultivars and management to enhance their stress tolerance to extreme climate conditions^6–16^. These advances include new breeding technologies (e.g., marker-assisted selection^13,17^, genomic selection^18,19^, and gene-editing^14,15,20,21^) and improved practices^22–25^. Some studies find, however, that current breeding programs are not preparing crop cultivars sufficiently for climatic variability^26^ and others find that crop cultivars adapted to extreme climate conditions might perform worse under other conditions. For instance, productivity of heat-resistant plants could be reduced under normal temperatures^27–30^ and drought-resistant cultivars can perform worse than conventional ones when sufficient water is available^31–33^. Studies analyzing the adaptability of heat and drought tolerance traits through laboratory and field experiments do not provide systematic evidence of the extent to which crops are becoming more resilient overall to climate conditions^16^ or whether the directions of climate-neutral technical change and climate adaptation efforts are synergistic or conflicting.

Large scale statistical studies^34–40^ have typically examined the responsiveness of crop yields to annual changes in climate variables but ignored the potential for climate-specific adaptation or climate-neutral technical change to mitigate the impacts of climate change. Exceptions include analysis of the role for climate-neutral technological change^41,42^ or adaptation to climate change^1^ but there has been no assessment of both using the same framework.

In Fig. 1, adaptation is illustrated by the ability to switch from a yield-climate relationship implied by Curve 1, under current conditions, to one that performs better with changed conditions (Curve 2). With adaptation, an increase in temperature from ${t}_{1}$ to ${t}_{2}$ lowers yield by ${V}_{0}-{V}_{1}$ instead of ${V}_{0}-{V}_{2}$. Burke and Emerick^1^ analyze the relationship between the yield difference at two distant points in time (i.e., ${V}_{0}-{V}_{1}$) and the difference in climate variables at those two points (i.e., ${{t}_{2}-t}_{1}$), which is reflected as the slope of segment $AB$. Unlike their long differences approach which implicitly restricts the responsiveness of yield to climate conditions at two points in time (slopes of Curve 1 at point $A$ and Curve 2 at point $B$) to be identical, we develop a flexible long differences approach to examine the extent to which corn and soybeans have adapted to climate that allows the responsiveness of yield to climate variables to vary across time (see ‘Methods’). For comparison, we also estimate the long differences approach and the fixed effects panel approach as in earlier studies^1,34–40^. We distinguish between normal and extreme values of heat (measured by growing degree days (GDD)) as well as of precipitation by constructing four climatic variables at growing season level: GDD between 0 and 29 °C (normal GDD), GDD above 29 °C (overheating GDD), precipitation below 42 cm (drought condition), and precipitation above 42 cm (normal precipitation), where growing season is defined as April 1 to September 30 in a year^1^. We disaggregate their effects on crop yield using data over 1951–2017 for 2,480 rain-fed counties that are east to the 100th meridian in the United States (see ‘Data’ and Supplementary Table S1). We also estimate the rate of climate-neutral technological change, which is the growth rate in yield after controlling for climate effects, and use the estimates to project the net effects of adaptation and climate change on crop yields by 2050 under a range of warming scenarios.

**Figure 1:**
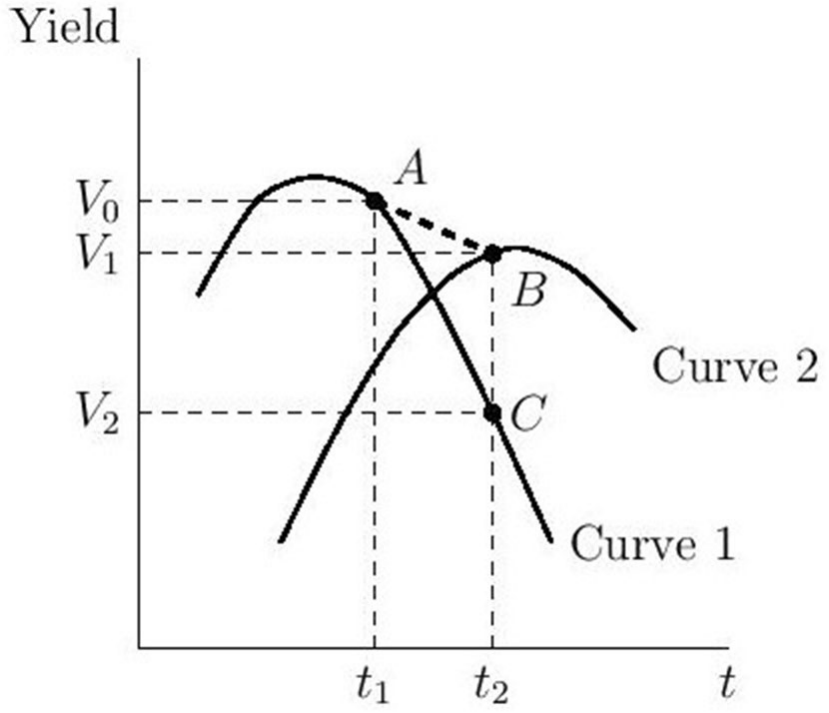
Yields of two crop cultivars as a function of a climate variable. *Notes*: Curves 1 and Curve 2 display the climate-yield relationships under cultivar 1 and cultivar 2, respectively. Point $A$ shows the yield level (${V}_{0}$) for Curve 1 when the climate variable is at value ${t}_{1}$. Similarly, points $B$ and $C$ show the yield levels (${V}_{1}$ and ${V}_{2}$, respectively) for Curve 2 and Curve 1, respectively, when the climate variable is at value ${t}_{2}$.

## Results

### Quantifying adaptation

**Figure 2:**
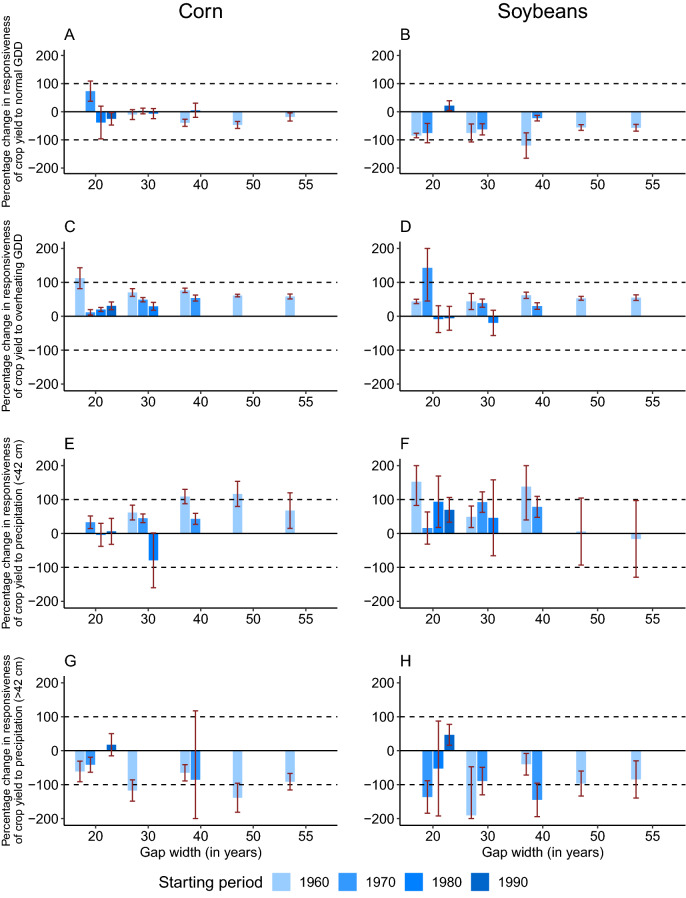
Percentage changes in responsiveness of crop yields to climate. *Notes*: This figure shows the percentage change in responsiveness of crop yield to climatic variables. We select two five-year periods with five different gap widths (i.e., 20-year, 30-year, 40-year, 50-year, and 55-year) and four different starting periods centered in 1960, 1970, 1980, and 1990 (in total 11 scenarios). For instance, a scenario with starting year as 1960 and 20-year gap width utilizes data over periods 1958–1962 and 1978–1982 to conduct the flexible long differences approach to estimate the climatic impact on yield over the two periods. The length of a bar represents the percentage change in responsiveness of crop yield to the corresponding climate variable over the two periods. In the context of Fig. 1, it is the difference between slope of Curve 1 at point $A$ and slope of Curve 2 at point $B$ (for computation details see ‘Methods’). A bar with a positive value indicates an enlargement of a positive responsiveness or a shrinkage in a negative responsiveness over the two periods, whereas a bar with a negative value indicates the opposite. The whiskers are 95% confidence intervals of the estimates. Some bars are missing because the estimated responsiveness to the corresponding climate variable in the earlier period is statistically insignificant, and thus, the percentage change is undefined. Graphs A, C, E, and G are for corn yield and Graphs B, D, F, and H are for soybean yield.

We measure the extent of adaptation to each of the four climate conditions by the ratio of ${{V}_{1}-V}_{2}$ to ${V}_{0}$ as demonstrated in Fig. 1, where ${{V}_{1}-V}_{2}$ is the avoided yield reduction due to adaptation and ${V}_{0}$ is initial yield level observed at ${t}_{1}$. We select two five-year periods that are 20-year, 30-year, 40-year, 50-year, and 55-year apart and examine the change in the relationship between crop yields and the four aforementioned climatic variables over the two periods using the flexible long differences approach (see ‘Methods’). Such a change in the relationship is viewed as adaptation if the change increases yield and as maladaptation if the change decreases yield. By choosing two far-apart periods we can analyze the effects of climate change in the medium to long run^1^. We analyze the extent of adaptation under 11 scenarios that differ in the widths of gap and the starting year (i.e., 1960, 1970, 1980, and 1990) (see Fig. 2 for the details of 11 scenarios). For instance, a scenario with starting year as 1960 and 20-year gap width utilizes data over periods 1958–1962 and 1978–1982 to conduct the flexible long differences analysis to estimate the extent of adaptation over the two periods.

We first analyze the change in the responsiveness of crop yields to climate over two periods. Reflected in Fig. 1, this is the extent to which the slope of Curve 1 at point $A$ and slope of Curve 2 at point $B$ differ. The results are presented in Fig. 2 (see ‘Methods’ for the details about calculations and see Supplementary Tables S2 and S3 for specific regression results). We find that among all 11 scenarios for the two crops and four climate variables, 68 out of the 88 (77%) estimates have a statistically significant difference in the responsiveness of yield to climate variable over the two periods, which suggests that adaptation or maladaptation is occurring. We find that the positive responsiveness of crop yields to normal GDD has declined over time (Graphs A and B) and the negative responsiveness to overheating GDD has also declined (Graphs C and D). In general, the scenarios with longer gap width are more likely to show this pattern. For instance, the corn yield loss caused by one additional overheating GDD has been reduced by 43.5% on average for the 20-year models and by 58.3% for the 55-year model because of adaptation. Similarly, the soybean yield loss caused by one additional overheating GDD has been reduced by 42.9% on average for the 20-year models and by 54.7% for 55-year model. While precipitation deficit has smaller negative impact on crop yields in the later period compared to decades ago, indicating increased drought tolerance (Graphs E and F), the positive responsiveness to normal precipitation has becomes smaller in the later period (Graphs G and H). These findings imply that although the two crops became more heat- and drought-tolerant over the past six decades, their capacity to benefit from the normal temperature and precipitation became lower over time.

**Figure 3:**
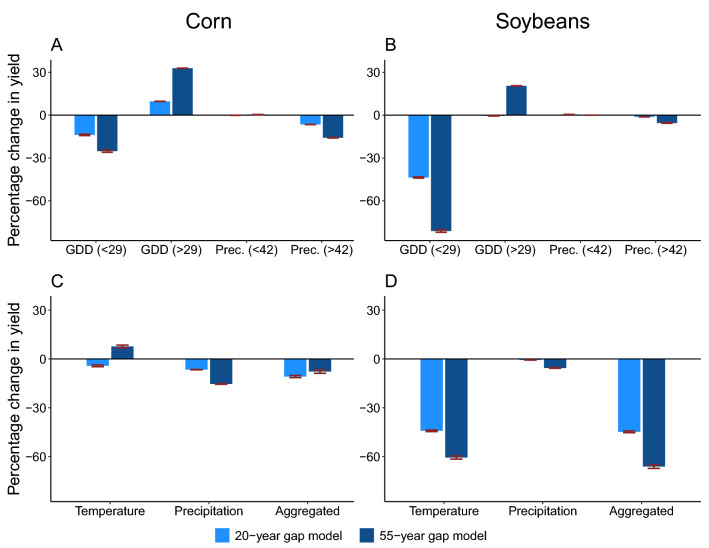
Partial and aggregated adaptation or maladaptation to climate change. *Notes*: GDD (< 29) represents the GDD between 0 and 29 °C and GDD (> 29) represents the GDD above 29 °C. Prec. (< 42) and Prec. (> 42) represent the precipitation below and above 42 cm, respectively. The daily climatic variables are aggregated to the growing season level, where the growing season is defined as April 1 to September 30 in a year. Graphs A and C are for corn yield and Graphs B and D are for soybean yield. The 20-year gap model uses 1978–1982 as the first period and 1998–2002 as the second period. The 55-year gap model uses 1958–1962 as the first period and 2013–2017 as the second period. The length of a bar shows the weighted average of all county-level partial or aggregated adaptation to the corresponding climate variables. It represents difference between the crop yields with and without adaption (e.g., the length of $BC$ in Fig. 1) divided by the initial crop yield (e.g., ${V}_{0}$ in Fig. 1). The negative values indicate maladaptation. The whiskers are 95% confidence intervals of the estimates. Values for Prec. (< 42) are close to 0. See Supplementary Table S5 for specific numerical values. To convert the change in log yield (say, $x$) to the percentage change in yield, we use the formula $\left({e}^{x}-1\right)\times 100\%$. Similar calculations are used for Figs. 4, 5, and Supplementary Fig. S1.

Next, we use the estimated statistical parameters (see Supplementary Tables S2 and S3) to conduct counterfactual analysis and then to quantify the partial adaptation of the two crops to the observed change in each of the four climate variables under each scenario. We then aggregate these estimates of partial adaptation to obtain an overall measure of adaptation over a given time period. For instance, the adaptation to overheating GDD is calculated by using the difference between the crop yields with and without adaptation to overheating GDD (i.e., the length of segment $BC$, or ${V}_{1}-{V}_{2}$, in Fig. 1) divided by the initial crop yield (i.e., ${V}_{0}$ in Fig. 1). If the result is negative, it implies maladaptation has reduced yield. The results of partial and aggregate adaptation are presented in two scenarios (over 1980–2000 and 1960–2015) are shown in Fig. 3; results under other scenarios are shown in Supplementary Fig. S1.

We find that the changes in yield due to climate variables are larger when the gap width is 55 years compared to 20 years, indicating that time-varying adaptation or maladaptation is more obvious in the longer-run (Fig. 3). We focus on results from the 55-year gap model (i.e., darker blue bars in Fig. 3) in the discussion hereafter. Adaptation to normal GDD is negative and statistically significant for both crops regardless of the scenario specifications (Graphs A and B). On average, over 1960–2015, maladaptation to normal GDD reduces the corn and soybean yields by 25.2% (about 0.76 metric ton/hectare, relative to average corn yield over 1958–1962) and 81.1% (about 1.19 metric ton/hectare, relative to average soybean yield over 1958–1962), respectively. On the other hand, in most cases, the adaptation to overheating GDD is positive and statistically significant, indicating that the two crops become more heat-resistant over time. On average, the adaptation to overheating GDD has increased corn and soybean yields by 32.8% (about 0.99 metric ton/hectare) and 20.5% (about 0.30 metric ton/hectare), respectively, over 1958–1962 levels. Overall, the adaptation to change in GDD in the past 55 years has increased corn yield by 7.7% (about 0.23 metric ton/hectare) but decreased soybean yield by 60.6% (about 0.89 metric ton/hectare) (Graphs C and D).

Adaptation to drought conditions (precipitation less than 42 cm) is small for both crops, although in most cases, statistically significant (see Graphs E and F in Supplementary Fig. S1), indicating that drought-tolerance has increased less than has heat-resistance, which is similar to the finding by Lobell et al.^43^ In contrast, adaptation to precipitation above 42 cm is negative and statistically significant, implying productivity under normal precipitation has become lower over time; this is consistent with the findings in Kahiluoto et al.^26^ that the current breeding programs and genomic selection fail to prepare crops for abundant rainfall and with those in Lobell et al.^43^ that corn yield has become more sensitive to soil water storage capacity. Maladaptation to precipitation above 42 cm has reduced corn and soybean yields by 15.8% (about 0.48 metric ton/hectare) and 5.5% (about 0.08 metric ton/hectare), respectively, in 2013–2017 period compared to yield in 1958–1962 (see Graphs A and B in Fig. 3). It follows that the aggregated adaptation to precipitation changes has decreased corn and soybean yields by 15.4% (about 0.50 metric ton/hectare) and 5.6% (about 0.08 metric ton/hectare), respectively, over 1960–2015 (see Graphs C and D in Fig. 3).

Considering both temperature and precipitation, we find that the adaptation in the past few decades has made both corn and soybeans more heat-resistant and drought-resistant, but reduced their productivity under normal temperature and precipitation. The yield-increasing effect of the enhanced stress resistance to both overheating GDD and drought has increased corn and soybean yields by 33% and 20%, respectively. This yield-increasing effect is smaller than the yield-decreasing effect due to maladaptation to normal GDD and precipitation above 42 cm, which decreases corn and soybean yields by 41% and 87%, respectively. As a result, the overall effect of the change in climate variables is a reduction in corn and soybean yields by 8% and 67%, respectively. This alarming finding is consistent with many early studies^1,26,43–46^. It reveals the complexity of climate change adaptation and indicates that current approaches to adaptation are not addressing the challenges of the multi-dimensional ways in which the climate is changing.

We estimate that climate-neutral technical change over 1960–2015 has increased corn and soybean yields by 1.47% and 3.92% annually. Earlier studies^41,42^ estimated these rates to be 1.44%-2.80% per year for corn and -0.08%-2.52% per year for soybeans over the 1960–2009 period. As a result, the predicted net increase in corn and soybean yields is 212% and 117% in 2013–2017 compared to 1958–1962, despite the maladaptation to climate condition. These predicted net increases in yields are close to observed increases by 223% and 110%, respectively, over this period.

### Projections of future climate change effects on yields

**Figure 4:**
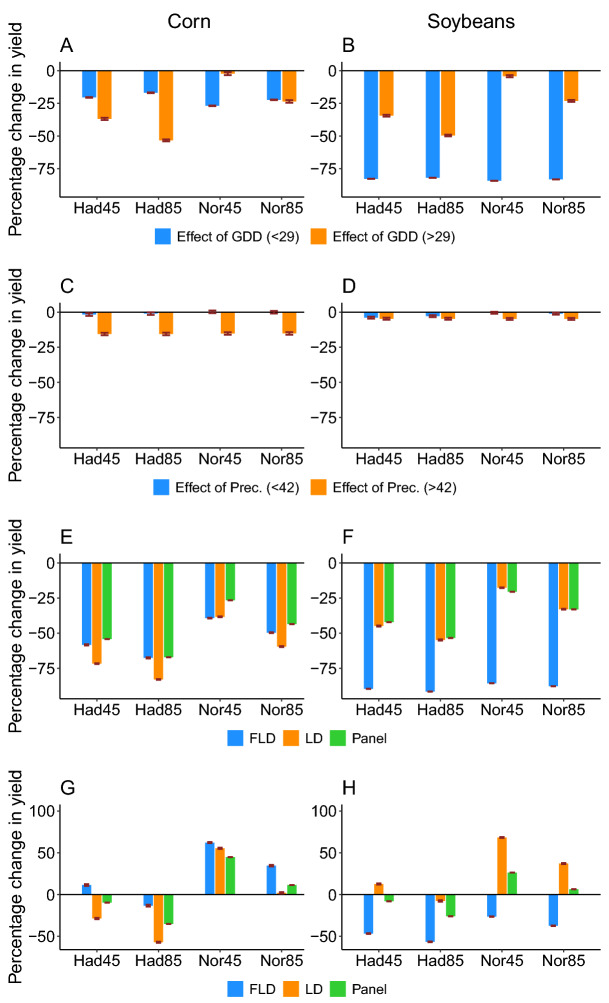
Projected future change of crop yields by 2050. *Notes*: Had45 is the abbreviation for the climate model HadGEM2-ES365 with warming scenario RCP 4.5. Similar interpretation applies to Had85, Nor45, and Nor85, where ‘Nor’ stands for global climate model NorESM1-M. FLD stands for the flexible long differences approach, LD for the long differences approach, and Panel for the fixed effects panel approach. The length of a bar represents the level of the predicted percentage change in yield and the whiskers are the 95% confidence intervals of the prediction. Graphs A and B show the projections using only temperature changes. Graphs C and D show the projections using only precipitation changes. Graphs E and F show the projected yield change caused by overall climate change under three model specifications: Flexible long differences (FLD) model, long differences (LD) model, and fixed effects panel data model (Panel). Graphs G and H show the overall projected yield change caused by climate change and by climate-neutral technological improvement under the aforementioned three model specifications. Graphs A, C, E and G are for corn yield and Graphs B, D, F and H are for soybean yield. We assume that the adaptation and the annual rate for climate-neutral technical change from 2015 to 2050 are the same as that from 1960 to 2015.

We construct projections of the potential impacts of climate change on corn and soybean yields in the future, by 2050, in the United States based on the results from our statistical analysis and using data from two global climate models (namely, HadGEM2-ES365 and NorESM1-M) for two warming scenarios (namely, Representative Concentration Pathway (RCP) 4.5 and RCP 8.5). For these projections, we assume that the changes in the marginal effects of climatic variables in the future are the same as they were in the past; the annual growth rate of crop yields caused by climate-neutral technical change in the future is the same as it was in the past; and the planting area and growing season in the US do not change over time. We utilize the most recent period in our sample (2013–2017) as the base period and 2048–2052 as the future period (see ‘Methods’ for details of projections, and see Supplementary Table S4 for the summary statistics of data in these two periods). Compared to the 2013–2017 period, the global climate models predict that overheating GDD in the 2048–2052 period will be significantly higher and that the precipitation will be lower. Our yield projections are based on the regression results from the statistical model for periods 1958–1962 and 2013–2017. Here, we implicitly assume that the magnitude of adaptation over the 2013–2017 period to the 2048–2052 period is the same as that over 1958–1962 to 2013–2017. We obtain projected yield changes for each county over 2048–2052 and present their weighted average in Fig. 4 using the county-level planted acreage in 2015 as the weight.

We find that although normal GDD (i.e., ‘GDD (< 29)’) over 2048–2052 is higher than that over 2013–2017 (see Supplementary Table S4), the reduced capacity to benefit from normal GDD over time will decrease corn yield by about 22% (average of blue bar values in Graph A of Fig. 4) over 2048–2052 when compared with that over 2013–2017. Despite increased heat tolerance, there is a negative and statistically significant effect of overheating GDD in all cases because 2048–2052 is much hotter than 2013–2017. Under the hottest warming scenario (i.e., HadGEM2-ES365 RCP 8.5), the increase in overheating GDD decreases corn and soybean yields by 53.3% (about 5.21 metric ton/hectare) and 49.6% (about 1.53 metric ton/hectare) relative to average soybean yield over 2013–2017), respectively (Graphs A and B in Fig. 4).

Changes in precipitation below 42 cm have a negligible impact on crop yields because the magnitude of change in this variable is small (see Supplementary Table S4). However, future change in precipitation above 42 cm has a negative and statistically significant effect on crop yield, regardless of the scenarios, because of maladaptation to abundant precipitation (above 42 cm). For corn, this negative impact is about 15% (average of orange bar values of Prec. (> 42) in Graph C of Fig. 4, about 1.47 metric ton/hectare in absolute values); while for soybeans, this effect is relatively smaller, at about 5% (average of orange bar values of Prec. (> 42) in Graph D of Fig. 4, about 0.15 metric ton/hectare in absolute values).

We estimated the projected crop yield changes caused by overall changes in climate variables based on regression results from three different statistical approaches: The flexible long differences approach (our preferred approach), the long differences approach, and the fixed effects panel approach (see ‘Method’ for the details of these approaches). All three approaches project that the future change in climate will significantly decrease crop yields (see Graphs E and F in Fig. 4). The flexible long differences approach predicts a reduction in corn and soybean yields by 39%-68% (about 3.8–6.6 metric ton/hectare) and 86%-92% (about 2.6–2.8 metric ton/hectare), respectively, in 2050 compared to 2013–2017, depending on warming scenario and global climate model used. The yield reduction for corn predicted by the flexible long differences approach is smaller than that under the long differences approach (see Graph E). The flexible long differences approach projects a larger yield reduction for soybeans than that projected by the long differences model and the fixed effects model (see Graph F). This indicates that without considering the time-varying responsiveness of crop yields to climate, there is significant potential to over- or under-estimate the impact of climate change on crop yields.

Assuming that climate-neutral technical change will continue to increase corn and soybean yields at the same annual rates as those over 1960–2015 (i.e., 1.47% and 3.92%, respectively), by 2050, the joint effect of climate change and climate-neutral technology will cause corn and soybean yields to decrease by 13% (about 1.27 metric ton/hectare) and 57% (about 1.75 metric ton/hectare), respectively, under the hottest warming scenario (i.e., HadGEM2-ES365 RCP 8.5). Under the mildest warming scenario (i.e., NorESM1-M RCP 4.5), this joint effect will cause corn yield to increase by 62% (about 6.06 metric ton/hectare) and soybean yield to decrease by 26% (about 0.80 metric ton/hectare). On average over the four warming scenarios, the joint effect will increase corn yield by 23.7% (about 2.32 metric ton/hectare) and decrease soybean yield by 41.7% (about 1.28 metric ton/hectare), by 2050 compared with the 2013–2017 levels (see Graphs G and H in Fig. 4).

**Figure 5:**
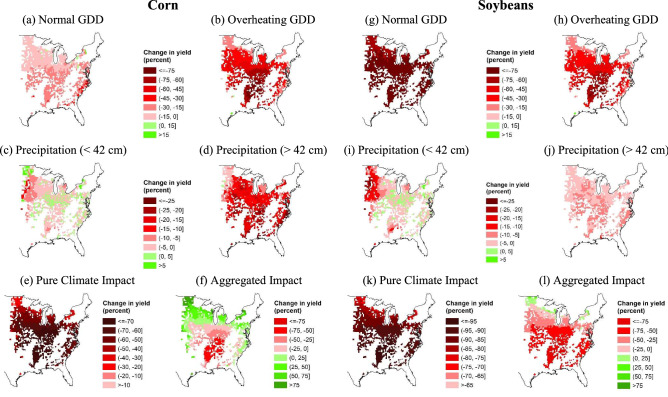
Projected crop yield changes under HadGEM2-ES365 RCP 8.5. *Notes*: Maps (**a**) to (**e**) (respectively, (**g**) to (**k**)) show the projected change in corn (respectively, soybean) yield caused by the changes of different climate variables and overall climate change by 2050 at county level compared to the average yield over 2013–2017. Maps (**f**) and (**l**) show the projected change in corn and soybean yields, respectively, caused by the aggregated impact including the climate change effect and the impact of climate-neutral technological changes. Maps (**a**), (**b**), (**g**), and (**h**) (respectively, (**c**), (**d**), (**i**), and (**j**)) share the same legend. All maps are generated using ArcGIS Desktop (v. 10.8.1).

Fig. 5presents the geographical pattern of the impact of climate change on crop yields across the US rain-fed counties projected using estimates from the flexible long differences approach under the 55-year gap scenario and under future climate predicted by HadGEM2-ES365 with RCP 8.5. The majority of counties in the central and southeastern of the United States show sharp declines in crop yields by 2050 due to future temperature changes and overall climate change, whereas some northern counties can expect an increase in crop yields. The regional differences in the impact of temperature change are large. Both the effects of the change in normal GDD and overheating GDD on crop yields range from lower than -75% to above 15% (see maps (a) and (b) for corn, (g) and (h) for soybeans in Fig. 5). Compared to the effect of temperature changes, the impact of precipitation change is relatively small and less diverse across counties (see maps (c) and (d) for corn, (i) and (j) for soybeans in Fig. 5), ranging between about -25% to about 10%. The pure climate impacts on corn and soybean yields range from below -80% to about -10% (see maps (e) and (k) in Fig. 5). After incorporating climate-neutral technological change, the net impact also shows large spatial variations with yield increases by about 50% in the North and decreases by more than 75% in the South (see maps (f) and (l) in Fig. 5).

## Discussion

A great deal of scientific effort is being dedicated to adapting crops to extreme climate conditions but there is little existing evidence on a large scale of the extent and type of conditions to which crops have adapted and the overall implications of climate change for crop yields under future warming scenarios after considering both climate-neutral and climate-related technological changes. This study develops a flexible long differences approach to examine the extent to which corn and soybeans in the US have adapted to various aspects of climate conditions over the last six decades and to assess the rate of climate-neutral technical change affecting crop yields.

The flexible long differences approach developed here makes several contributions to quantifying the effectiveness of adaptation to climate change compared to the long differences and fixed effects model. First, it recognizes that the responsiveness of crop yield to climate is changing over two periods that are far apart. Second, it disaggregates the adaptation of crops to multiple dimensions of climate conditions and shows the trade-offs generated by the current methods of adapting crops to climate change. Third, it quantifies the rate of climate-neutral technological change after controlling for the impact of changes in climate conditions and climate-related adaptation.

We find that both crops have become more heat and drought tolerant, but these improvements have been achieved at the cost of reducing crop productivity under normal GDD and normal precipitation conditions. Climate-neutral yield increased at the rate of 1.47% per year for corn and 3.92% per year for soybeans between period 1958–1962 and period 2013–2017, and partly offset the declines caused by climate change. Continuing this pattern of adaptation to climate will significantly reduce corn and soybean yields by 39%-68% and 86%-92%, respectively, due to climate change by 2050, depending on the warming scenario. Despite this, if annual rates of climate-neutral technical change over 1960–2015 continue, then corn yield can be expected to change by -13% to 62%, whereas soybean yield will decrease by 26%-57% in 2050, compared with 2013–2017 levels, depending on the warming scenario.

These findings are significant for two reasons. First, they show that scientific efforts need to adapt crops not only for extreme heat and drought conditions but to be productive under a wider range of climate conditions. Second, they raise concern about our potential to avoid food insecurity in the next 30 years, as demands for food increase by 70%-100% by 2050^47^, without expanding land under crop production.

## Methods

Our flexible long differences approach builds on the fixed effects panel approach^34–39^ and the long differences approach^1^. The fixed effects panel approach applies panel data to examine the relationship between annual agricultural outcomes and annual weather using models as:

1 $${y}_{it}={\beta }_{0}+{\varvec{\beta}}{{\varvec{z}}}_{{\varvec{i}}{\varvec{t}}}+{c}_{i}+{\epsilon }_{it},$$

where $y$ is the agricultural outcome (e.g., yield, profits, or land value), ${\varvec{z}}$ a vector of the explanatory variables such as climate variables and time trend, $c$ the fixed effects that captures time-invariant, local characteristics of a county such as precipitation gradient and geographical characteristics, and $\epsilon$ the error term. Subscripts $i$ and $t$ stand for county $i$ and year $t$, respectively. In this study $y$ is the logarithm of crop yield. Although this approach mitigates the concern about omitted variable bias by controlling for the fixed effects and by using exogenous variation in annual weather, it has been criticized for failing to reflect farmers’ long-run responses to climate change. The long differences approach in Burke and Emerick^1^ takes two $n$-year periods denoted as period $a$ (an earlier period) and period $b$ (a more recent period) and computes the county level average crop yield in each period as $\overline{{y}_{i\tau }}=\frac{1}{n} {\sum }_{t\in \tau }{y}_{it}$ where $\tau \in \{a, b\}$. Average climate variables $\overline{{z}_{ia}}$ and $\overline{{z}_{ib}}$ are obtained similarly. Based on Eq. (1), we have:

2 $$\overline{{y}_{i\tau }}={\beta }_{0}+{\varvec{\beta}}\overline{{{\varvec{z}}}_{{\varvec{i}}{\varvec{\tau}}}}+{c}_{i}+\overline{{\epsilon }_{i\tau }}.$$

Taking difference over the two periods, Burke and Emerick^1^ develop a long differences approach that can be written as:

3 $$\Delta \overline{{y}_{i}}={\varvec{\beta}}\Delta \overline{{{\varvec{z}}}_{{\varvec{i}}}}+\Delta \overline{{\epsilon }_{i}}.$$

Notice that Eq. (3) is equivalent to analyzing Eq. (2) as a panel data fixed effects model^48^.

We extend these approaches by developing a flexible long differences approach to estimate the changes in the responsiveness of crop yields to climate over time. Specifically, in our empirical model, we add a period dummy in Eq. (2) and allow the period dummy to interact with climate variables. The interaction terms differentiate the responsiveness of crop yields to climate across the two periods and thereby mark the time-varying agricultural adaptations over time. Specifically, the flexible long differences model can be written as:

4 $$\overline{{y}_{i\tau }}={\beta }_{0}+{\eta }_{0}{D}_{\tau }+{\varvec{\beta}}\overline{{{\varvec{z}}}_{{\varvec{i}}{\varvec{\tau}}}}+{\varvec{\eta}}{(D}_{\tau }\times \overline{{{\varvec{z}}}_{{\varvec{i}}{\varvec{\tau}}}})+{c}_{i}+\overline{{\epsilon }_{i\tau }},$$

where ${D}_{\tau }$ is the period dummy variable with ${D}_{\tau }=0$ if $\tau =a$ and ${D}_{\tau }=1$ if $\tau =b$. We consider four climate variables, which are normal growing degree days (GDD) (i.e., GDD between 0 and 29 °C), overheating GDD (i.e., GDD over 29 °C), precipitation below 42 cm, and precipitation above 42 cm (see ‘Data’ for more details about these variables). Therefore, Eq. (4) can be written as:

5 $$\overline{{y}_{i\tau }}={\beta }_{0}+{\eta }_{0}{D}_{\tau }+{\beta }_{1}{GDD}_{i\tau ;0:29}+{\beta }_{2}{GDD}_{i\tau ;29+}+{\beta }_{3}{Prec}_{i\tau ;p<42}+{\beta }_{4}{Prec}_{i\tau ;p>42}+{\eta }_{1}\left({D}_{\tau }\times {GDD}_{i\tau ;0:29}\right)+{\eta }_{2}\left({D}_{\tau }\times {GDD}_{i\tau ;29+}\right)+{\eta }_{3}\left({D}_{\tau }\times {Prec}_{i\tau ;p<42}\right)+{\eta }_{4}\left({D}_{\tau }\times {Prec}_{i\tau ;p>42}\right)+{c}_{i}+\overline{{\epsilon }_{i\tau },}$$

where ${GDD}_{i\uptau ;0:29}$ and ${GDD}_{i\uptau ;29+}$ represent the average growing degree days for county $i$ in period $\tau$ when the temperature interval is $[0, 29]$ and $(29, \infty )$, respectively; ${Prec}_{i\uptau ;p<42}$ and ${Prec}_{i\uptau ;p>42}$ represent the average growing season precipitation below and above a given precipitation threshold 42 cm respectively for county $i$ in period $\tau$.

We apply Eq. (5) to analyze the impact of climate change on corn and soybean yields using the aforementioned eleven scenarios (see Supplementary Tables S2 and S3). One can see that in Supplementary Tables S2 and S3, most of the estimates for interaction terms between period dummy and climate variables are statistically significant, which suggests that the impact of climate on crop yields is changing over time. Fig. 2 depicts the estimated ${\eta }_{k}/{\beta }_{k}$, $k\in \left\{\mathrm{1,2},\mathrm{3,4}\right\}$, which compares the magnitude of adaptation along four dimensions. For instance, ${\beta }_{2}$ measures the responsiveness of crop yield to overheating GDD in the early period whereas ${\beta }_{2}+{\eta }_{2}$ measures the responsiveness of crop yield to overheating GDD in the later period. By computing the ratio ${\eta }_{2}/{\beta }_{2}$, we quantify the change in the responsiveness between the two periods. Numerically, the length of each bar in Fig. 2 equals $\mathrm{sign}(\eta )\times |\eta /\beta |\times 100\%$, where $\mathrm{sign}(\eta )$ indicates the direction of the change in responsiveness. Some missing bars in Fig. 2 suggest that the estimated responsiveness to the corresponding climate variable in the earlier period ($\beta$) is statistically insignificant, and thus, the percentage change is undefined.

The potential violations to the identification assumption in Eq. (5) are omitted variables such as local emissions and local land-use change. The local emissions could influence both climate and crop yields while the local land-use change could be an adaptation to climate change, indicating that the land-use change and climate change are correlated^1^. However, as discussed in Burke and Emerick^1^, these omitted variable concerns are limited. First, any indirect effect of aerosols from local emissions on crops through temperature and precipitation has already been reflected in the model. The concern about whether the effect of aerosols on solar radiation has significant impact on crop yield is more relevant, because solar radiation benefits crop productivity. However, as Burke and Emerick^1^ point out, such effect is ambiguous because on the one hand, aerosols reduce direct beam radiation that reaches the top part of a plant, and on the other hand, they increase diffused sunlight that can reach the lower part of a plant. Moreover, there is no evidence that significant variation in local emissions exists. Second, according to Burke and Emerick^1^, little change in land area or land management practices occurred that can influence climate and crop yield simultaneously. Recent scientific evidence also suggests that the differential warming trends in the United States over the past several decades are mainly caused by ocean temperature changes^1,49^. Therefore, we believe that Eq. (5) is identified.

Quantifying the effect of climate change on crop yields using the empirical model in Eq. (5) has substantial advantages compared to previous studies. First, the flexible long differences approach assumes a time-varying relationship between agricultural production and climate variables by allowing the climate variables in the two periods to have different marginal effects on crop yields. Second, Eq. (2) implies that Burke and Emerick^1^ may suffer from omitted variable bias because the interaction terms ${(D}_{\tau }\times \overline{{{\varvec{z}}}_{{\varvec{i}}{\varvec{\tau}}}})$ are left in the error term. Third, unlike the fixed effects panel approach that captures the short-run adjustment and the long differences approach that quantifies long-run adaptation by comparing the long-run responsiveness obtained from the long differences approach to the short-run adjustment obtained from the fixed effects panel approach, the flexible long differences approach directly estimates the long-run agricultural adaptation to climate change, that is, the parameters ${\eta }_{1}$ to ${\eta }_{4}$ in Eq. (5). Thus, the flexible long differences approach can provide a more coherent estimation of long-run adaptation than do the previous studies.

The partial adaptation results presented in Fig. 3 and Supplementary Table S5 are calculated by $({\eta }_{k}\times \overline{{z}_{ib}})$ where $k\in \{\mathrm{1,2},\mathrm{3,4}\}$, which represents multiplying the changes in the yield impact of the corresponding climate variable (i.e., ${\eta }_{k}$ ) with the climate variable in period $b$ (the later period) (i.e., $\overline{{z}_{ib}}$). For example, the adaptation to overheating GDD is given by ${\eta }_{2}\times {GDD}_{ib;29+}$. To convert the change in log yield to the percentage change in yield, we apply the following procedure. Let $\stackrel{\sim }{{y}_{b}}$ be the weighted average of crop yield in the base period and $x$ be estimated change in log yield. Then, the approximate percentage change in yield is $\frac{{e}^{\mathrm{log}\left(\stackrel{\sim }{{y}_{b}}\right)+x}-\stackrel{\sim }{{y}_{b}}}{\stackrel{\sim }{{y}_{b}}}\times 100\%=\frac{{e}^{\mathrm{log}\left(\stackrel{\sim }{{y}_{b}}\right)}{e}^{x}-\stackrel{\sim }{{y}_{b}}}{\stackrel{\sim }{{y}_{b}}}\times 100\%=\frac{\stackrel{\sim }{{y}_{b}}{e}^{x}-\stackrel{\sim }{{y}_{b}}}{\stackrel{\sim }{{y}_{b}}}\times 100\%=\left({e}^{x}-1\right)\times 100\%$.

The annual growth rate of crop yields caused by climate-neutral technical change over a given period is ${[({e}^{{\eta }_{0}}-1)}^{1/n}-1]\times 100\%$, where ${\eta }_{0}$ is the coefficient for ${D}_{\tau }$ in the corresponding model [see Eq. (4)] and $n$ is the gap width. The predicted percentage change in crop yield in 2013–2017 compared to 1958–1962 is $\left({e}^{\Delta \overline{{y}_{i}}}-1\right)\times 100\%$, where $\Delta \overline{{y}_{i}}={\eta }_{0}+{\varvec{\beta}}\Delta \overline{{{\varvec{z}}}_{{\varvec{i}}}}+{\varvec{\eta}}\overline{{{\varvec{z}}}_{{\varvec{i}}{\varvec{b}}}}$, where $\Delta \overline{{{\varvec{z}}}_{{\varvec{i}}}}$ is a vector of the change in climate variables over 1960–2015 and is $\overline{{{\varvec{z}}}_{{\varvec{i}}{\varvec{b}}}}$ a vector of climate variables in 2013–2017. The observed percentage change in crop yield is $\frac{\overline{{y}_{ib}}-\overline{{y}_{ia}}}{\overline{{y}_{ia}}}$, where $\overline{{y}_{i\tau }}$ is the average yield level in period $\tau \in \{a, b\}$.

The projection for future climate impact on crop yields is conducted separately based on results from the flexible long differences approach [i.e., Eq. (5)], the long differences approach [i.e., Eq. (3)], and the fixed effects panel approach [i.e., Eq. (1)]. For the flexible long differences approach and the long differences approach, we first analyze the yield climate relationship between the 1958–1962 period and the 2013–2017 period using the two approaches and obtain the estimated coefficients. We then choose 2013–2017 as the first period and 2048–2052 as the second period for the projections. We first compute the differences in the five-year average climate variables between the two periods and denote them as ${\Delta GDD}_{i;0:29}$, $\Delta {GDD}_{i;29+}$, $\Delta {Prec}_{i;p<42}$, and ${\Delta Prec}_{i;p>42}$. Then, the projected change in log yield due to climate change using the flexible long differences approach is:

6 $$\Delta \overline{{y}_{i}}=\widehat{{\beta }_{1}}{\Delta GDD}_{i;0:29}+\widehat{{\beta }_{2}}\Delta {GDD}_{i;29+}+\widehat{{\beta }_{3}}\Delta {Prec}_{i;p<42}+\widehat{{\beta }_{4}}{\Delta Prec}_{i;p>42}+\widehat{{\eta }_{1}}{GDD}_{ib;0:29}+\widehat{{\eta }_{2}}{GDD}_{ib;29+}+\widehat{{\eta }_{3}}{Prec}_{ib;p<42}+\widehat{{\eta }_{4}}{Prec}_{ib;p>42}.$$

Similarly, using the long differences approach, the projected value is:

7 $$\Delta \overline{{y}_{i}}=\stackrel{\sim }{{\beta }_{1}}{\Delta GDD}_{i;0:29}+\stackrel{\sim }{{\beta }_{2}}\Delta {GDD}_{i;29+}+\stackrel{\sim }{{\beta }_{3}}\Delta {Prec}_{i;p<42}+\stackrel{\sim }{{\beta }_{4}}{\Delta Prec}_{i;p>42}.$$

The climate-neutral technological advances are reflected by the estimate of ${\eta }_{0}$ in the flexible long differences approach [see Eq. (5)] and the estimate of ${\beta }_{0}$ in the long differences approach [see Eq. (2)]. We assume that the annual rate of climate-neutral technological over 2015–2050 (35 years) to be identical to that over 1960–2015 (55 years). Based on this assumption, by using ${\eta }_{0}^{^{\prime}}=\mathrm{log}[{\left({e}^{{\eta }_{0}}-1\right)}^\frac{35}{55}+1]$ and ${\beta }_{0}^{^{\prime}}=\mathrm{log}[{\left({e}^{{\beta }_{0}}-1\right)}^\frac{35}{55}+1]$ we obtain the climate-neutral technological advances in the future 35 years for the flexible long differences approach and long differences approach, respectively. We then add the climate-neutral technological advances to the projected change in log yield caused by climate change to obtain the overall projected change in log yield.

Our findings differ from the projections in Burke and Emerick^1^ because the flexible long differences approach in this study provides a more coherent estimation of adaptation within one model instead of comparing between the results from long differences model and those from fixed effects panel model as that in Burke and Emerick^1^.

For the fixed effects panel approach, we first analyze the yield climate relationship in the 1951–2017 period using the balance panel dataset and obtain the estimated coefficients. Similar to the other two approaches, we choose 2013–2017 and 2048–2052 as the two periods for the projections and compute the differences in the five-year average climate variables between the two periods. Using the same notations as above, the projected change in log yield due to climate change using the fixed effects panel approach is:

8 $$\Delta \overline{{y }_{i}}={ {\overset{\lower0.5em\hbox{$\smash{\scriptscriptstyle\smile}$}}{\beta }}_{1}}{\Delta GDD}_{i;0:29}+{ {\overset{\lower0.5em\hbox{$\smash{\scriptscriptstyle\smile}$}}{\beta }}_{2}}\Delta {GDD}_{i;29+}+{ {\overset{\lower0.5em\hbox{$\smash{\scriptscriptstyle\smile}$}}{\beta }}_{3}}\Delta {Prec}_{i;p<42}+{ {\overset{\lower0.5em\hbox{$\smash{\scriptscriptstyle\smile}$}}{\beta }}_{4}}{\Delta Prec}_{i;p>42}.$$

Because the two periods are 35 years apart, the climate-neutral technological advances are represented by $$35\times { {\overset{\lower0.5em\hbox{$\smash{\scriptscriptstyle\smile}$}}{\beta }}_{time}}$$, where $${ {\overset{\lower0.5em\hbox{$\smash{\scriptscriptstyle\smile}$}}{\beta }}_{time}}$$ is the coefficient of the time trend which is included in vector $$\varvec{z}$$ in Eq. (1). We include the climate-neutral technological advances when we consider the impact of climate-neutral technology improvement. Similar to the calculation of partial adaptation, for all the three approaches, we convert the change in log yield to the percentage change in yield using the formula $$\left({e}^{x}-1\right)\times 100\%$$, where $$x$$ is estimated change in log yield.

## Data

The crop yield and planted acreage data are obtained from the U.S. Department of Agriculture’s (USDA) National Agricultural Statistics Service (NASS)^50^. The data are available at county-year level. Supplementary Fig. S2 shows the sample average yields of corn and soybeans from 1951 to 2017. One can see that in the past six decades, the average corn yield has increased considerably with obvious fluctuations largely caused by extreme climate conditions. The average soybean yield also increases, but the growth rate is much smaller and the fluctuations are less obvious compared to corn. The climate data are obtained from Schlenker and Roberts^35^, where they build the dataset based on the Parameter-elevation Regressions on Independent Slopes Model (PRISM) weather dataset. The extended dataset up to 2017 is available on Schlenker’s website (http://www.columbia.edu/~ws2162/links.html). Daily values for precipitation, minimum temperatures, and maximum temperatures for each county are available over 1951–2017.

Existing studies have found strong evidence that the relationship between temperature and agricultural outcomes, such as crop yield, is nonlinear^1,35^. To capture such nonlinearity, we employ the widely used concept of growing degree days (GDD), which is a measurement of heat accumulation and reflects the amount of time that the temperature falls in a given interval on a specific day. We follow Schlenker and Roberts^35^ and Burke and Emerick^1^ to apply the formula developed by Snyder^51^ to obtain the county level daily GDD value and aggregate these daily values over a fixed growing season to obtain the annual normal GDD and overheating GDD for each county given different temperature intervals. Following Burke and Emerick^1^, the temperature interval is 0 to 29 degrees Celsius for the normal GDD and above 29 degrees Celsius for the overheating GDD. We set the growing season to be April 1 to September 30 following Burke and Emerick^1^. Supplementary Table S1 includes the summary statistics of these variables.

Precipitation variables are computed in a similar way to those in Burke and Emerick^1^. We first subtract a threshold ${p}_{0}$ from the growing season precipitation to obtain the difference ($\delta$) between the actual precipitation and the threshold. Then we let ‘precipitation below threshold’ equal $\mathrm{max}\{-\delta , 0\}$ and let ‘precipitation above threshold’ equal $\mathrm{max}\{\delta ,0\}$. Following Burke and Emerick^1^, the precipitation threshold is set to be ${p}_{0}=$ 42 cm, defined as a threshold under which water deficiency would occur. It is close to the tenth percentile of annual county precipitation (i.e., 41.3 cm) and grants the lowest sum of squared residuals among all possible values of ${p}_{0}$^1^. Therefore, variable “precipitation lower than 42 cm” measures the severity of water deficiency. The larger the value is, the severer the deficiency. Following Burke and Emerick^1^, when we conduct regression analysis using the flexible long differences approach or long differences approach, we exclude counties that produce corn or soybeans in only one period.

The predicted future climate data over 2048–2052 are from two global climate models—HadGEM2-ES365 and NorESM1-M, where HadGEM2-ES365 predicts warmer future climate and NorESM1-M predicts cooler future climate. Two warming scenarios—RCP 4.5 and RCP 8.5—are used to mark the medium and the warmest scenarios in each climate prediction model. Supplementary Table S4 includes the summary statistics of the future climate data.

## Supplementary Information

Supplementary Information.
