# Supplementary material for: Maladaptation of U.S. corn and soybeans to a changing climate
Source: Sci Rep. 2021 Jun 11;11:12351. doi: 10.1038/s41598-021-91192-5 (PMC8196191; doi:10.1038/s41598-021-91192-5)
Supplement: Supplementary file 1 — Supplementary Information. [file 41598_2021_91192_MOESM1_ESM.pdf]

Supplementary Information for

Maladaptation of U.S. Corn and Soybeans to a Changing Climate

Chengzheng Yu[1], Ruiqing Miao[2], Madhu Khanna[1]*

[1] Department of Agricultural and Consumer Economics, University of Illinois at Urbana-Champaign, Urbana, IL, USA

[2] Department of Agricultural Economics and Rural Sociology, Auburn University, Auburn, AL, USA

* Corresponding author: Madhu Khanna (khanna1@illinois.edu)

Table S1: Summary Statistics of Variables (1951-2017)

Variable	Mean	SD	Min	Max
Corn Yield (in metric ton per hectare)	5.29	2.64	0.00	15.49
Soybean Yield (in metric ton per hectare)	1.98	0.74	0.05	4.92
GDD (<29℃)	3,647.49	521.97	2,088.50	7,008.09
GDD (>29℃)	61.41	56.89	0.00	552.32
Precipitation (<42 cm) (in cm)	0.66	2.52	0.00	36.59
Precipitation (>42 cm) (in cm)	19.47	15.50	0.00	147.18
Growing Season Precipitation (in cm)	60.81	16.50	5.41	189.18

Notes: The average yields of corn and soybeans are obtained from the U.S. Department of Agriculture's (USDA) National Agricultural Statistics Service (NASS). The climate data are drawn from Schlenker and Roberts (2009), where they build the dataset based on the Parameter-elevation Regressions on Independent Slopes Model (PRISM) weather dataset.

Table S2: Estimates of the Impacts of Temperature and Precipitation on US Corn Yields

Period	1960-1980	1960-1990	1960-2000	1960-2010	1960-2015	1970-1990	1970-2000	1970-2010	1980-2000	1980-2010	1990-2010
	(1)	(2)	(3)	(4)	(5)	(6)	(7)	(8)	(9)	(10)	(11)
Period Dummy	0.7508	0.6957	1.5156	1.7795	1.1714	-0.4528	0.2774	0.4136	0.4179	0.5764	0.6328
	(0.0684)	(0.0828)	(0.1038)	(0.1153)	(0.1061)	(0.0770)	(0.1053)	(0.1411)	(0.0809)	(0.0947)	(0.0842)
GDD (<29℃)	0.0000	0.0003	0.0007	0.0007	0.0004	0.0003	0.0007	0.0004	-0.0001	-0.0004	-0.0004
	(0.0000)	(0.0001)	(0.0001)	(0.0001)	(0.0001)	(0.0001)	(0.0001)	(0.0001)	(0.0000)	(0.0000)	(0.0001)
GDD (>29℃)	-0.0024	-0.0066	-0.0101	-0.0149	-0.0113	-0.0058	-0.0082	-0.0080	-0.0067	-0.0045	-0.0039
	(0.0005)	(0.0005)	(0.0006)	(0.0006)	(0.0007)	(0.0004)	(0.0007)	(0.0008)	(0.0005)	(0.0005)	(0.0006)
Precipitation (<42 cm)	-0.0011	-0.0272	-0.0508	-0.0323	-0.0597	-0.0382	-0.0789	-0.0825	-0.0279	-0.0208	-0.0209
	(0.0050)	(0.0052)	(0.0056)	(0.0056)	(0.0075)	(0.0050)	(0.0065)	(0.0080)	(0.0042)	(0.0051)	(0.0032)
Precipitation (>42 cm)	0.0037	0.0058	0.0072	0.0050	0.0071	0.0055	-0.0008	-0.0023	0.0002	-0.0017	-0.0049
	(0.0007)	(0.0009)	(0.0011)	(0.0011)	(0.0013)	(0.0008)	(0.0012)	(0.0015)	(0.0010)	(0.0011)	(0.0009)
D_τ × GDD (<29℃)	-0.0001	0.0000	-0.0003	-0.0003	-0.0001	0.0002	0.0000	0.0000	0.0000	0.0000	-0.0001
	(0.0000)	(0.0000)	(0.0000)	(0.0000)	(0.0000)	(0.0000)	(0.0000)	(0.0000)	(0.0000)	(0.0000)	(0.0000)
D_τ × GDD (>29℃)	0.0026	0.0047	0.0077	0.0091	0.0066	0.0007	0.0040	0.0043	0.0013	0.0013	0.0012
	(0.0002)	(0.0003)	(0.0003)	(0.0004)	(0.0004)	(0.0002)	(0.0003)	(0.0005)	(0.0002)	(0.0003)	(0.0003)
D_τ × Preci (<42 cm)	0.0434	0.0167	0.0552	0.0376	0.0402	0.0126	0.0353	0.0356	-0.0011	-0.0165	0.0013
	(0.0041)	(0.0046)	(0.0062)	(0.0065)	(0.0137)	(0.0048)	(0.0069)	(0.0093)	(0.0047)	(0.0055)	(0.0042)
D_τ × Preci (>42 cm)	-0.0023	-0.0068	-0.0047	-0.0069	-0.0065	-0.0023	-0.0033	-0.0020	-0.0039	-0.0056	0.0009
	(0.0006)	(0.0008)	(0.0011)	(0.0011)	(0.0011)	(0.0007)	(0.0011)	(0.0014)	(0.0008)	(0.0010)	(0.0009)
Number of counties	1840	1682	1528	1390	1184	1798	1624	1464	1639	1485	1482
Adjusted R squared	0.85	0.88	0.88	0.91	0.95	0.68	0.68	0.71	0.34	0.56	0.45

Notes: Numbers in the parenthesis are standard errors. Regressions are weighted by the crop planted acreage in the middle year of the starting period. For example, the regression for 1960-1980 model is weighted by the crop planted acreage in 1960.

Table S3: Estimates of the Impacts of Temperature and Precipitation on US Soybean Yields

Period	1960-1980	1960-1990	1960-2000	1960-2010	1960-2015	1970-1990	1970-2000	1970-2010	1980-2000	1980-2010	1990-2010
	(1)	(2)	(3)	(4)	(5)	(6)	(7)	(8)	(9)	(10)	(11)
Period Dummy	1.7893	1.5934	2.6936	2.2953	2.2284	1.1720	1.7164	1.0191	0.7515	0.1146	0.0317
	(0.0857)	(0.1031)	(0.1024)	(0.1184)	(0.0985)	(0.0791)	(0.0905)	(0.1084)	(0.0635)	(0.0791)	(0.0855)
GDD (<29°C)	0.0005	0.0005	0.0005	0.0009	0.0008	0.0004	0.0006	0.0006	0.0000	0.0000	-0.0003
	(0.0000)	(0.0001)	(0.0001)	(0.0001)	(0.0001)	(0.0001)	(0.0001)	(0.0001)	(0.0000)	(0.0000)	(0.0001)
GDD (>29°C)	-0.0062	-0.0031	-0.0060	-0.0092	-0.0082	-0.0013	-0.0036	-0.0050	-0.0008	-0.0013	-0.0020
	(0.0005)	(0.0005)	(0.0006)	(0.0006)	(0.0007)	(0.0004)	(0.0006)	(0.0006)	(0.0003)	(0.0004)	(0.0005)
Precipitation (<42 cm)	-0.0178	-0.0250	-0.0126	-0.0140	-0.0333	-0.0194	-0.0289	-0.0320	-0.0087	-0.0072	-0.0219
	(0.0059)	(0.0064)	(0.0064)	(0.0064)	(0.0073)	(0.0053)	(0.0061)	(0.0069)	(0.0032)	(0.0045)	(0.0028)
Precipitation (>42 cm)	-0.0001	0.0021	0.0054	0.0040	0.0026	0.0035	0.0042	0.0043	-0.0012	-0.0004	-0.0040
	(0.0009)	(0.0010)	(0.0012)	(0.0011)	(0.0012)	(0.0007)	(0.0010)	(0.0010)	(0.0007)	(0.0009)	(0.0007)
D_τ × GDD (<29°C)	-0.0005	-0.0003	-0.0007	-0.0005	-0.0005	-0.0003	-0.0004	-0.0001	-0.0002	0.0001	0.0001
	(0.0000)	(0.0000)	(0.0000)	(0.0000)	(0.0000)	(0.0000)	(0.0000)	(0.0000)	(0.0000)	(0.0000)	(0.0000)
D_τ × GDD (>29°C)	0.0027	0.0014	0.0037	0.0049	0.0045	0.0019	0.0014	0.0015	-0.0001	-0.0002	-0.0001
	(0.0003)	(0.0004)	(0.0004)	(0.0004)	(0.0003)	(0.0003)	(0.0003)	(0.0003)	(0.0002)	(0.0002)	(0.0003)
D_τ × Preci (<42 cm)	0.0272	0.0123	0.0174	0.0008	-0.0054	0.0031	0.0267	0.0251	0.0081	0.0033	0.0152
	(0.0056)	(0.0065)	(0.0076)	(0.0073)	(0.0193)	(0.0054)	(0.0070)	(0.0079)	(0.0040)	(0.0050)	(0.0043)
D_τ × Preci (>42 cm)	0.0005	-0.0040	-0.0022	-0.0039	-0.0022	-0.0047	-0.0038	-0.0062	-0.0007	-0.0017	0.0019
	(0.0009)	(0.0010)	(0.0011)	(0.0011)	(0.0010)	(0.0007)	(0.0009)	(0.0010)	(0.0006)	(0.0008)	(0.0008)
Number of counties	1108	1055	987	980	856	1261	1123	1066	1380	1291	1333
Adjusted R squared	0.72	0.74	0.85	0.89	0.94	0.45	0.69	0.78	0.37	0.62	0.47

Notes: Numbers in the parenthesis are standard errors. Regressions are weighted by the crop planted acreage in the middle year of the starting period. For example, the regression for 1960-1980 model is weighted by the crop planted acreage in 1960.

Table S4: Summary Statistics of Projection Climate Variables

Baseline Period	Variable	Mean	SD	Min	Max
	GDD (<29°C)	3,571.55	436.05	2,433.83	4,638.70
	GDD (>29°C)	38.75	35.25	0.00	199.93
	Precipitation (<42 cm)	0.11	0.52	0.00	5.57
	Precipitation (>42 cm)	26.64	9.41	0.00	72.04
**Prediction Model**	**Climate Variable**	**Mean**	**SD**	**Min**	**Max**
Had RCP 4.5	GDD (<29°C)	3,762.94	356.86	2,918.42	4,699.41
	GDD (>29°C)	177.54	82.92	29.12	401.82
	Precipitation (<42 cm)	1.26	2.13	0.00	10.08
	Precipitation (>42 cm)	14.89	5.71	0.00	36.74
Had RCP 8.5	GDD (<29°C)	3,887.87	328.37	2,996.76	4,737.29
	GDD (>29°C)	236.87	116.36	38.36	539.28
	Precipitation (<42 cm)	0.94	1.66	0.00	9.84
	Precipitation (>42 cm)	14.75	7.02	0.72	37.91
Nor RCP 4.5	GDD (<29°C)	3,530.24	363.49	2,798.71	4,463.17
	GDD (>29°C)	92.70	56.06	12.52	254.56
	Precipitation (<42 cm)	0.23	1.00	0.00	7.58
	Precipitation (>42 cm)	20.66	8.98	0.00	53.20
Nor RCP 8.5	GDD (<29°C)	3,705.70	384.08	2,785.69	4,688.70
	GDD (>29°C)	142.54	77.46	15.25	415.05
	Precipitation (<42 cm)	0.38	1.10	0.00	7.93
	Precipitation (>42 cm)	20.99	10.93	0.28	67.20

Notes: The baseline period is 2013-2017 period. Had RCP 4.5 is the abbreviation for the climate model HadGEM2-ES365 with warming scenario RCP 4.5. Similar interpretation applies to Had RCP 8.5, Nor RCP 4.5, and Nor RCP 8.5, where 'Nor' indicates NorESM1-M. The dataset includes county-year level predicted GDD and precipitation from 2048 to 2052.

Table S5: Estimated Partial and Aggregated Adaptation to Climate Change

	20-year gap<br>Corn<br>(1)	20-year gap<br>Soybeans<br>(2)	55-year gap<br>Corn<br>(3)	55-year gap<br>Soybeans<br>(4)
Adaptation to GDD (<29°C)	-13.83	-43.66	-25.19	-81.11
Lower bound of 95% confident interval	-14.32	-44.08	-25.99	-81.97
Upper bound of 95% confident interval	-13.33	-43.23	-24.38	-80.25
Adaptation to GDD (>29°C)	9.63	-0.49	32.85	20.51
Lower bound of 95% confident interval	9.56	-0.55	32.77	20.42
Upper bound of 95% confident interval	9.70	-0.43	32.93	20.60
Adaptation to Temperature Changes	-4.20	-44.15	7.66	-60.60
Lower bound of 95% confident interval	-4.76	-44.63	6.78	-61.55
Upper bound of 95% confident interval	-3.63	-43.66	8.55	-59.65
Adaptation to Precipitation (<42 cm)	-0.09	0.53	0.40	-0.03
Lower bound of 95% confident interval	-0.11	0.51	0.39	-0.04
Upper bound of 95% confident interval	-0.07	0.54	0.41	-0.02
Adaptation to Precipitation (>42 cm)	-6.50	-1.17	-15.79	-5.54
Lower bound of 95% confident interval	-6.58	-1.23	-15.95	-5.72
Upper bound of 95% confident interval	-6.43	-1.11	-15.62	-5.36
Adaptation to Precipitation Changes	-6.60	-0.64	-15.39	-5.57
Lower bound of 95% confident interval	-6.69	-0.72	-15.56	-5.76
Upper bound of 95% confident interval	-6.50	-0.57	-15.21	-5.38
Aggregated Adaptation	-10.79	-44.79	-7.72	-66.17
Lower bound of 95% confident interval	-11.45	-45.35	-8.79	-67.31
Upper bound of 95% confident interval	-10.14	-44.23	-6.66	-65.03

Notes: The 20-year gap model uses 1978-1982 as the first period and 1998-2002 as the second period. The 55-year gap model uses 1958-1962 as the first period and 2013-2017 as the second period. The value shows the weighted average (and the corresponding 95% confidence interval) of all county-level partial or aggregated adaptation to the corresponding climate variables. It represents difference between the crop yields with and without adaption (e.g., the length of BC in Figure 1) divided by the crop yield without this partial adaptation (e.g., V_0 in Figure 1). The negative values indicate maladaptation. To convert the change in log yield (say, x) to the percentage change in yield, we use the formula $(e^x - 1) \times 100\%$.

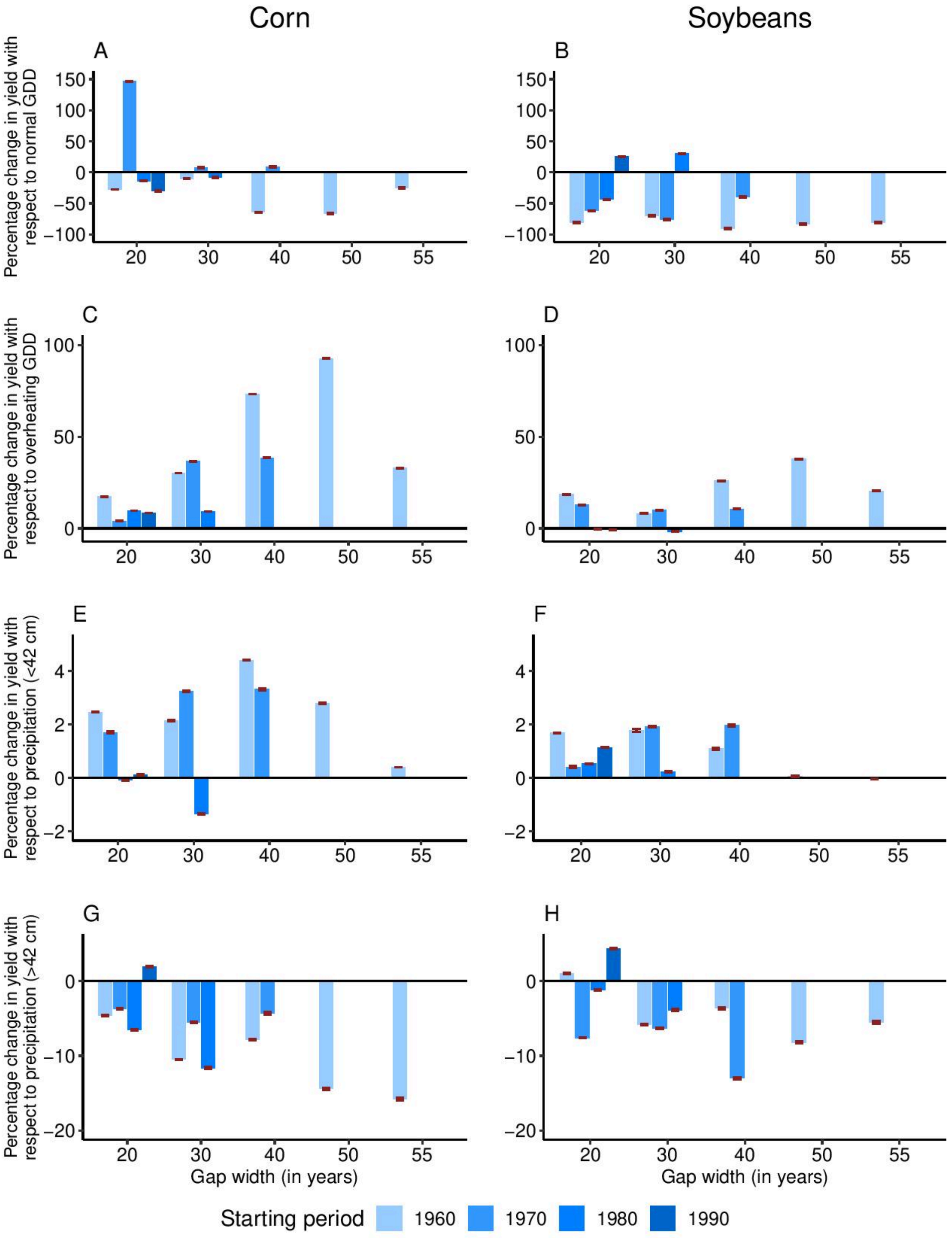

Figure S1: Partial Adaptation or Maladaptation to Climate Change

Notes: Graphs A, C, E, and G are for corn yield and Graphs B, D, F, and H are for soybean yield. The length of a bar shows the weighted average of all county-level partial adaptation to the corresponding climate variable. It represents difference between the crop yields with and without adaption (e.g., the length of BC in Figure 1) divided by the crop yield without this partial adaptation (e.g., V_0 in Figure 1). The negative values indicate maladaptation. The whiskers are 95% confidence intervals of the estimates. To convert the change in log yield (say, x) to the percentage change in yield, we use the formula $(e^x - 1) \times 100\%$.

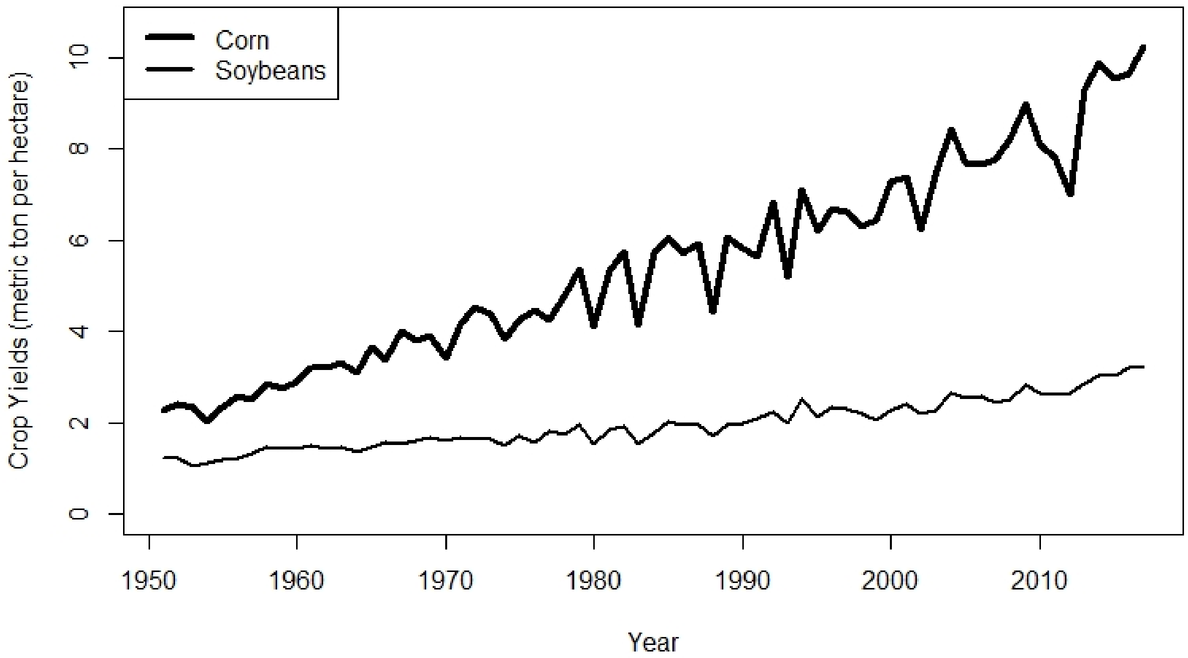

Figure S2: Average Yields of U.S. Corn and Soybeans from 1951 to 2017
Note: The average yields of corn and soybeans are obtained from the U.S. Department of Agriculture's (USDA) National Agricultural Statistics Service (NASS).
